# The influence of social networks on finding and selecting healthcare professionals

**DOI:** 10.1590/1807-3107bor-2025.vol39.001

**Published:** 2025-01-13

**Authors:** Letícia Miquelitto GASPARONI, Vinícius Neves MARCOS, Cláudio Mendes PANNUTI, Sílvia Maria Morales PEREIRA

**Affiliations:** (a) Universidade Federal de Juiz de Fora – UFJF, School of Dentistry, Department of Dental Clinic, Juiz de Fora, MG, Brazil.; (b) Universidade Federal de Juiz de Fora - UFJF, University Hospital, Department of Diagnostic Imaging, Juiz de Fora, MG, Brazil.; (c) Universidade de São Paulo – USP, School of Dentistry, Department of Stomatology, São Paulo, SP, Brazil.; (d) Universidade Estadual de Campinas – Unicamp, School of Applied Sciences, Campinas, SP, Brazil.

**Keywords:** Social Networking, Social Media, Health, Health Personnel

## Abstract

Social networks consist of a group of individuals connected by family, work, or other interests and facilitated by an online structure or platform. They are also a relatively recent and widely used marketing phenomenon that is constantly evolving. The healthcare field includes professions such as social work, biology, biomedicine, physical education, nursing, pharmacy, physiotherapy, speech therapy, medicine, veterinary medicine, nutrition, dentistry, psychology, and occupational therapy. The present study aimed to analyze the influence of social networks in the process of finding and selecting healthcare professionals. The methodology was a survey using a structured questionnaire created on Google Forms. Descriptive research was carried out with non-probabilistic convenience and snowball sampling, followed by quantitative data analysis. A total of 268 participants who signed the informed consent were included in the study. The findings revealed that the most common way to find healthcare professionals is through recommendations from friends/relatives and other professionals, followed by the use of social networks. The majority of participants used social networks to search for healthcare professionals, with Instagram being the most widely used platform. Sponsored advertisements can be an effective way to reach potential new patients. The most valued characteristics in healthcare content creators were reliability, expertise, and the ability to convey messages, with health-related explanations in an easily understandable manner being the preferred type of content. Therefore, this study revealed that social networks can influence the search for and selection of healthcare professionals.

## Introduction

According to the Brazilian Institute of Geography and Statistics (IBGE),^1^ 82.7% of Brazilian households have access to the internet, with mobile phones being the most commonly used device for internet access. With the changes that the internet and access to information have brought to the population, the way people seek healthcare services and professionals has also evolved.

*Homo sapiens* is a social animal.^2^ Throughout history, humanity has thrived in social communities where members shared knowledge, opinions, and experiences, strengthened by a sense of belonging. As technology continues to advance, the definition of a social network also evolves. A social network can be defined as a network of individuals (such as friends, acquaintances, and colleagues) connected by interpersonal relationships, along with an online service or website through which people create and maintain interpersonal relationships.^3^ There has been a proliferation of social network platforms in recent years, such as WhatsApp, Instagram, Facebook, TikTok, Messenger, Kuaishou, Telegram, Pinterest, Twitter, LinkedIn, Skype, Snapchat, Discord, iMessage, Reddit, among others, leading to an increasing usage of these networks in Brazil and worldwide.^4^

In 2021, Farsi^5^ published a literature review on the use of social networks worldwide as an essential tool in the healthcare field. The author used a combination of the following keywords for the search: “Social Media, Social Network, Internet, WhatsApp, Instagram, Facebook, YouTube, Twitter, LinkedIn, Health, Medicine, Medical, Dentistry, Nursing, Telemedicine, Recruitment, Education, Career, Behavior, and Research”. This review provided an overview of the role of social networks in improving direct patient care, increasing public awareness, facilitating research, connecting healthcare professionals, enhancing medical education, and combating public health crises.

According to the National Health Council, health professions include social work, biology, biomedicine, physical education, nursing, pharmacy, physiotherapy, speech therapy, medicine, veterinary medicine, nutrition, dentistry, psychology, and occupational therapy.^6^ The success of social networks has led many professionals to use these tools, which is perfectly legal as long as the principles of the respective council’s Code of Ethics are respected.

In 2018, Chan and Leung^7^ conducted a systematic review on the use of social networks for communication among healthcare professionals for: a) clinical practice; b) professional networking; and c) education and training to identify areas for future research in health communication. The authors observed that all studies included in the review reported that social networks improved effective communication and information sharing. However, it was also noted that the use of social networks is limited by the need for technical skills, data protection concerns, privacy, and responsibility, as well as issues related to professionalism. The authors concluded that the evolving use of social networks requires robust research to explore their full potential and relative effectiveness in professional communication.

Rukavina, et al.^8^ conducted a review on the dangers and benefits of social networks in the e-professionalism of healthcare professionals. In line with the evolution of the use of social network worldwide, their popularity has also extended to healthcare professionals. The review included 88 studies with participants from various healthcare professions. Three main benefits were identified: a) professional networking and collaboration; b) professional education and training; and d) patient education and health promotion. Five recognized dangers were identified for the selected studies: a) loosening of accountability; b) compromising confidentiality; c) confusing professional boundaries; d) displaying unprofessional behavior; and e) legal issues and disciplinary consequences. The review highlighted existing recommendations to include e-professionalism in the educational curricula of healthcare professionals. It also provided new insights and guidelines for future research in this area, as there is a clear need for robust research to investigate emerging social networks platforms, the efficiency of guidelines and educational interventions, and the specificities of each profession regarding their potential and use of social networks.

Social networks are a relatively recent and widely used marketing strategy and are constantly evolving. It is known that there are demographic differences in user profiles of each platform.^9^ However, to date, there is no research demonstrating the influence of social networks on the search and selection of healthcare professionals in Brazil.

Therefore, the aim of this study was to analyze the use and influence of social networks in the process of searching and selecting healthcare professionals.

## Methods

This study was conducted following the recommendations of Marconi and Lakatos.^10^ It is a descriptive research^11^ using non-probabilistic convenience and snowball sampling, followed by quantitative data analysis^12^.

A survey was conducted using a structured questionnaire created on the Google Forms platform. The questionnaire was applied in Brazil during the month of August, 2022, and distributed through the following social network platforms: WhatsApp, Instagram, and Facebook.

The questionnaire was in Portuguese, and consisted of three parts: the first part included the informed consent form (ICF) and the screening question “Do you follow or accompany any healthcare professional on social networks?”; the second part contained questions related to participant characteristics without disclosing their identity; and the third part focused on specific issues related to the use and influence of social networks in the process of finding and selecting healthcare professionals. The questionnaire was developed based on the studies by Malhotra.^11^ The complete questionnaire and the raw data collected can be found on the Open Science Framework (OSF) platform through the following link: https://osf.io/zdn8t/?view_only=1a6c67f04d67454bb491b320df756c16.

The following inclusion criteria were applied: 18 years of age or older and affirmative answer to the screening question. Refusal to sign the ICF was used as an exclusion criterion.

Since this was a survey of public opinion with anonymous participants, there was no need to submit the research for review by the Research Ethics Committee, in accordance with Brazilian Resolution CNS No. 510, dated April 7, 2016.

## Results

A total of 279 participants agreed to participate in the research by signing the informed consent form, while one participant refused. The target population consisted of individuals who followed healthcare professionals on social networks. After the screening question, 268 participants completed the survey and 11 participants did not fit the target population of the study and were excluded.

Of the 268 participants (Table 1), 215 (80.2%) were female. The majority were within the age range of 29 to 39 years (42.2%), followed by 18 to 28 years (32.8%). The most prevalent level of education was completed post-graduation (specialization, MBA) (31.7%), followed by complete undergraduate education (23.5%). The proportion of single participants was very similar to that of married participants (47.8% vs. 47.4%, respectively). The majority were within the income range of R$1,213.00 to R$3,637.00 (22.6%), followed by R$13,338.00 or higher (22.2%), R$3,638.00 to R$6,062.00 (18.4%), R$6,063.00 to R$8,487.00 (15%), up to R$1,212.00 (8.3%), R$8,488.00 to R$10,912.00 (6.8%), and R$10,913.00 to R$13,337.00 (6.8%). The participants lived in the following Brazilian regions: Northeast (48%), Southeast (38.8%), South (7.1%), Central-West (3.8%), and North (2.3%).

**Table 1:** Demographic data of participants.

Variables	n	%
Sex		
Female	215	80.2
Male	53	19.8
Age group (years)		
18–28 years	88	32.8
29–39	113	42.2
40–50	47	17.5
51–61	10	3.7
> 62	10	3.7
Education level		
Incomplete Elementary School	1	0.4
Complete Elementary School	3	1.1
Incomplete High School	1	0.4
Complete High School	42	15.7
Incomplete College/University	42	15.7
Complete College/University	63	23.5
Specialization, MBA	85	31.7
Master’s, Doctorate	31	11.6
Marital status		
Single	128	47.8
Married	127	47.4
Divorced/Separated	12	4.5
Not declared	1	0.4
Monthly family income		
Up to R$1,212.00	22	8.3
R$1,213.00 to R$3,637.00	60	22.6
R$3,638.00 to R$6,062.00	49	18.4
R$6,063.00 to R$8,487.00	40	15
R$8,488.00 to R$10,912.00	18	6.8
R$10,913.00 to R$13,337.00	18	6.8
Above R$13,338.00	59	22.2
Brazilian region		
Central-West	10	3.8
Northeast	129	48
North	6	2.3
Southeast	104	38.8
South	19	7.1
**Total**	**268**	**100**

**Figure 1:**
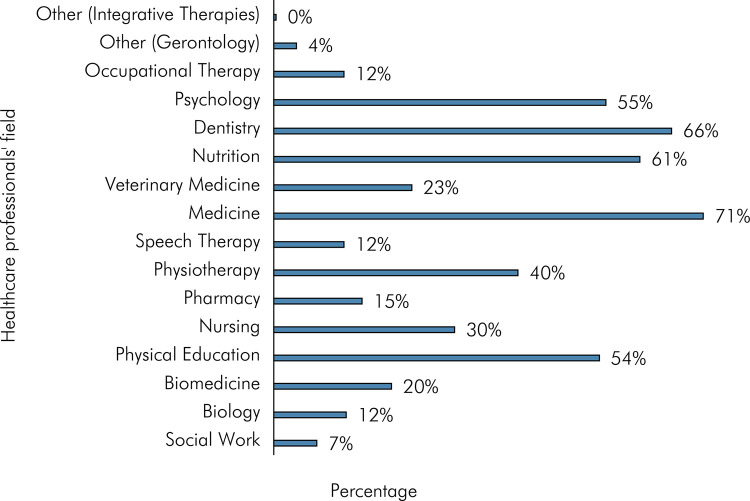
Healthcare professionals’ field that the research participants follow or accompany on social networks.

The first question of the second block complemented the screening question, aiming to determine the healthcare fields of the professionals that the participants followed or supported on social networks (Figure 1). For this question, participants could select multiple options. As shown in Figure 1, the healthcare field with the most followers/supporters was medicine (71.2%), followed by dentistry (65.9%), nutrition (60.7%), psychology (55.1%), physical education (53.9%), physiotherapy (40.4%), nursing (30%), veterinary medicine (22.8%), biomedicine (19.5%), pharmacy (14.6%), biology (12%), speech therapy (11.6%), occupational therapy (11.6%), and social work (7.1%). Under the “other” option, 3.8% of participants specified gerontology and 0.4% specified integrative therapies.

**Figure 2:**
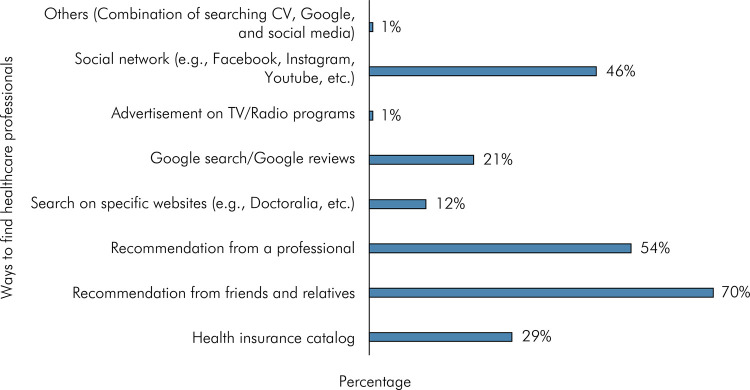
Methods used by research participants to find healthcare professionals.

When asked “What methods do you most commonly use to find healthcare professionals?”, participants could select up to three options. The majority of participants (70.4%) indicated “recommendation from friends and relatives” as their top method, followed by “professional recommendation” (53.6%), and “social network” (46.4%) (Figure 2).

When asked “Have you ever searched for a healthcare professional on social network platforms (e.g., Facebook, Instagram, YouTube, among others)?”, the majority of participants responded affirmatively (80.1%) (Table 2), and most (85.3%) used Instagram to search for healthcare professionals. When asked “Is the number of followers on Instagram important to you when deciding on a healthcare professional?”, the majority of participants responded negatively (65.9%). When asked if they had used hashtags to search for healthcare professionals on social networks, 80.2% of respondents stated they had not. To the question “Have you become a patient of a healthcare professional you found through social networks?”, the majority of participants responded affirmatively (54.3%). Table 2 also shows the social network platforms where participants who answered “yes” to the previous question found the healthcare professional(s) they became patients of; 60.1% of participants found these professionals on Instagram. When asked “Have you encountered healthcare professionals through sponsored advertising on social network?”, the majority of participants responded positively (56.8%). Finally, to the question “Regarding the statement: I would become a patient of a healthcare professional I encountered through sponsored advertising on social networks”, the majority of participants responded neither agree nor disagree (41.1%), followed by agree (39.9%), and strongly agree (11.4%) (Table 2).

**Table 2:** Data on the process of finding and selecting healthcare professionals.

Variables	%
Have you searched for any healthcare professional on social networks (e.g., Facebook, Instagram, YouTube, among others)?	
Yes	80.1
No	19.9
If yes, which social network platforms have you searched to find healthcare professionals?	
Instagram	85.3
Facebook	15.1
WhatsApp	12.2
YouTube	10.9
LinkedIn	6.3
TikTok	0.8
Messenger	0.8
Other (Lattes Platform)	0.8
Other (Doctoralia)	0.4
Is the number of followers on Instagram important to you when choosing a healthcare professional?	
Yes	34.1
No	65.9
Do you use/have you used hashtags (#) to search for healthcare professionals on social networks?	
Yes	19.8
No	80.2
Have you become a patient of any healthcare professional you met through social networks?	
Yes	54.3
No	45.7
If yes, through which social networks platforms did you meet this healthcare professional?	
Instagram	60.1
Facebook	1.8
WhatsApp	3.6
YouTube	0.4
Other (I used to validate professional conduct)	0.4
Other (Google)	0.4
Have you encountered healthcare professionals through sponsored advertisements on social networks?	
Yes	56.8
No	43.2
Regarding the statement: “I would become a patient of a healthcare professional I met through sponsored advertisements on social networks.”	
Strongly disagree	3.8
Disagree	3.8
Neither agree nor disagree	41.1
Agree	39.9
Strongly agree	11.4

**Figure 3:**
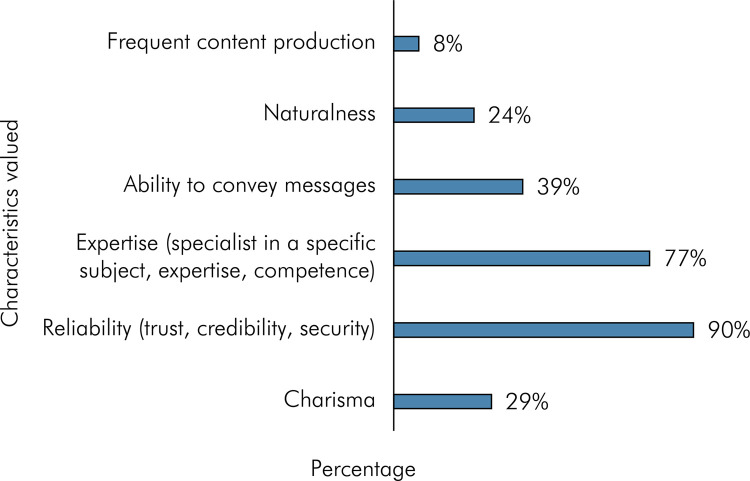
Characteristics valued in a healthcare content creator.

**Figure 4:**
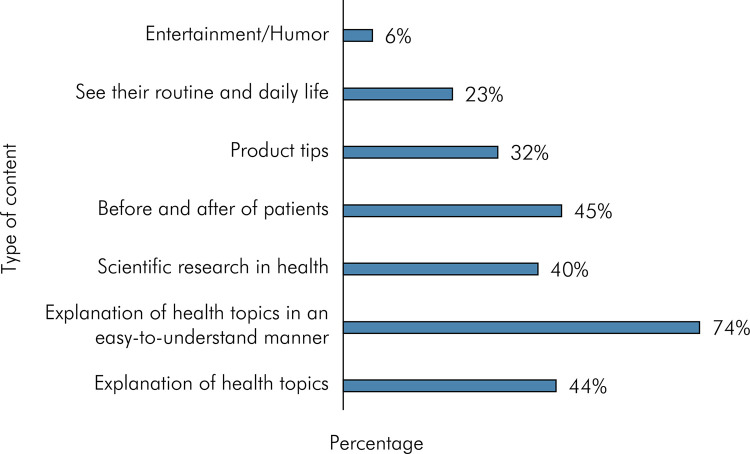
Type of content produced by healthcare professionals that interests the most on social networks.

When asked, “What characteristics do you value in a healthcare content creator?”, participants could select up to three options. The majority of participants (89.8%) selected the option “Reliability,” followed by “Expertise” (76.7%), and “Ability to convey the message” was the third most selected option (38.7%), as revealed in Figure 3. Thus, when asked, “What type of content produced by healthcare professionals interests you the most on social networks?”, participants could select up to three options. As shown in Figure 4, the majority of participants (73.7%) selected the option “Explanation of health topics in an easy-to-understand manner”, followed by “Before and after patient cases” (45.1%), and “Explanation of health topics” (44%).

## Discussion

According to IBGE,^13^ women account for 51.1% of the country’s population. There is no significant difference in gender nationally, but in the North region there is a higher proportion of men. The “Digital 2022 Global Overview Report” published specific data for Brazil.^14^ It was reported that 171.5 million Brazilians use social networks, which corresponds to 79.9% of the total population, with 54.7% being female and 45.3% being male. There was a 14.3% increase in social networks usage compared to the previous year, 2021. Regarding the demographic profile, data from Facebook, Instagram, and Messenger showed that most users are of the age group of 25-34 years (28%), followed by 18–24 years (22%), 35–44 years (20.1%), 45–54 years (12.3%), 55–64 years (7.3%), 13–17 years (5.7%), and + 65 years (4.3%). Compared to our data, there is similarity regarding the age groups that most use social networks, despite the different age group classifications used in the two studies. The data from the Digital 2022 report also showed a higher number of female users in all age groups. Although this study was designed in a different format than the Digital 2022 survey and the questionnaire was shared on social networks, our data also indicates a higher participation of females.

In Brazil, women are also the main users of healthcare services, both for their own needs and to accompany children, elderly individuals, persons with disabilities, and others. Women are not only more present in social networks, but also play a central role in caring for and maintaining family health, and tend to be more concerned and seek health information more frequently. This highlights the importance of producing clear, accessible, and reliable health content on social networks to meet the demands of this majority of users.

Regarding the level of education, the results obtained in this study differ from the data reported by IBGE,^1^ where the education level of individuals aged 25 years or older is classified as follows: no education (6.4%), incomplete primary education (32.2%), complete primary education (8%), incomplete secondary education (4.5%), complete secondary education (27.4%), incomplete higher education (4%), and complete higher education (17.4%). These discrepancies arise due to the convenience sampling used in this study. Nevertheless, the National Household Sample Survey (PNAD) conducted by IBGE^13^ showed that the average monthly per capita household income was R$ 1,353.00, and most of the participants in this research fall within this monthly family income range.

Health literacy refers to the ability of individuals to “gain access to, understand, and use information in ways which promote and maintain good health” for themselves, their families, and their communities.^15^ The results of this research suggest that users from different income levels use social networks to seek health information and find healthcare professionals. Socioeconomic status may influence how users access health information and interact on social networks, but the widespread availability of smartphones and internet access mitigate these disparities. The economic impact on health literacy may vary. Individuals with higher income and education may have more resources to recognize reliable information and make informed decisions regarding health. However, this is not inevitable. One limitation of the present study is that it did not measure health literacy. This seems to be an underexplored research area and future studies on this topic could provide interesting insights regarding social networks.

The demographic characteristics of the participants indicate a diverse and representative sample of the general population, with a wide range of age groups, educational backgrounds, marital status, and income levels. The distribution of participants across different regions of the country also ensures a broader perspective on the use of social networks in relation to healthcare professionals. The predominance of female participants aligns with previous studies that have shown women to be more active users of social network platforms.^16^ The high percentage of participants aged between 18 and 39 years indicates that social networks use among healthcare consumers is particularly prevalent among younger generations. Another limitation of this study is related to the Brazilian region. The raw data collected contain information about the Brazilian state in which each participant resides. Although participants from all Brazilian regions were included, the Northeast region had a much higher representation from the state of Bahia. This was influenced by convenience sampling and snowball sampling, in which participants are encouraged to recruit other participants from their acquaintances, and an influencer from the state of Bahia managed to recruit many other participants. However, this is not expected to affect our overall conclusions, but extrapolation to other states or regions might be affected, especially in the Northeast, due to selection bias.

Figure 1 shows greater searches for medicine, dentistry, and nutrition compared to other health professionals on social networks. This likely occurred because these health fields have higher public interest or demand for information. People often seek advice or second opinions about medical conditions, dental issues, or nutritional guidance. Additionally, people frequently search for symptoms, treatment options, dietary advice, and oral health tips on the internet because these issues directly impact their quality of life. However, other areas are also important and have a lot of content to be explored.

The dissemination of products and services among people is known as word-of-mouth advertising. Over 80% of individuals follow the recommendations from family members, friends, or professionals when acquiring a product or service^17^, and this was also revealed in this study. However, it is worth noting that despite recommendation from friends and relatives or professionals being ranked first and second, respectively, social networks ranked third, demonstrating the potential of this tool to reach potential patients if used strategically (Figure 2).

A total of 85.3% of the participants in this research used Instagram to search for healthcare professionals (Table 2). These data corroborate the findings of the “Digital 2022: Brazil” survey,^14^ where 90.1% of participants indicated Instagram as the most used social network platforms. In the “Digital 2022: Brazil” survey, WhatsApp ranked first (96.4%). Boulos, et al.^18^ provided an overview of the various applications of Instagram and WhatsApp in the healthcare field. They reported that smartphones are effective in a variety of social learning contexts, healthcare communication, and medical care, including patient care, monitoring, rehabilitation, communication, diagnosis, teaching, and research. The authors also highlighted the use of Instagram and WhatsApp as timely tools for communication among healthcare teams. However, caution is advised when searching for healthcare professionals on social networks platforms.

It is also noteworthy that the “Plataforma Lattes” (Lattes Platform) was mentioned by 0.8% of participants under the “other” option. Lattes Platform is a Brazilian curriculum database system, created and maintained by the National Council for Scientific and Technological Development (CNPq), which integrates curriculum databases, research groups, and institutions into a single information system.^19^ Searching for healthcare professionals on the Lattes Platform is straightforward and provides an in-depth understanding of the professional’s background and qualifications. This result might indicate that people do not search for or consult professionals on the Lattes platform due to a lack of knowledge about the platform and its purpose. Federal strategies could be implemented to promote awareness of this platform, and professionals should always keep their curriculum up to date.

For 65.9% of the participants in this research, the number of followers on Instagram is not important when choosing a healthcare professional (Table 2). This may indicate that social network users are more interested in the quality of content and the credibility of the professional rather than their popularity or status on social network. It is believed that health influencers have a different profile than influencers in other niches. Figure 3 helps us understand this issue, showing that reliability is highly valued because it is believed to build trust, credibility, and security among the audience. This suggests that these are the most valued characteristics in healthcare professionals, rather than the number of followers. Social influence refers to a phenomenon where people are influenced by the opinions, beliefs, and attitudes of others. When applied to behavior, the influence of others has been considered the most prevalent factor in consumer decision-making^20^. Online platforms have led to the emergence of a new type of influencer: the digital influencer. These content creators have amassed a solid follower base^21^. The activities of digital influencers provide their followers with insights into their lives, experiences, and opinions. Digital influencers have been considered a kind of micro-celebrity. However, unlike traditional celebrities, digital influencers are likely to be more relatable as they share their personal lives and interact directly with their followers. Thus, digital influencers are perceived as more accessible and intimate. Consequently, their posts may foster the illusion of a personal relationship, which is likely to make consumers more susceptible to influence.^20^

In recent years, social networks have reshaped the way individuals interact by providing new means to develop relationships and stay socially connected and facilitating more reciprocal and continuous interactions among individuals, regardless of time and location^22^. According to Lee et al.,^23^ Instagram users have five primary social and psychological motives: social interaction, archiving, self-expression, escapism, and voyeurism, but these motives are not always devoid of negative consequences. One of these negative experiences is the fear of missing out (FoMO). FoMO usually refers to the concern of social network users of missing out on opportunities when they are offline or unable or unwilling to connect and communicate with others to the extent they desire.^22^ As a result, people are increasingly staying connected. The internet also allows anonymous access to a vast amount of specific information and opinions, which is reflected in an increase in health concerns or anxiety. Health information found online prompts individuals to seek consultations with professionals, leading to increased use of healthcare resources. In this context, the term cyberchondria emerges from “cyber,” referring to internet use, and “hypochondria,” referring to pathological health anxiety. Cyberchondria is the belief or fear of having a serious illness, often without a corresponding medical condition^24^. These and many other psychological issues cannot be overlooked by professionals who want to produce content on social networks, always considering the responsible use of these platforms.

Staying connected on social networks, however, does not provide a competitive advantage anymore because everyone is on social networks^20^. In this context, the use of certain strategies can make healthcare professionals stand out, including the use of sponsored advertisements. People can often be suspicious when the content comes from advertising. However, this research showed that 56.8% of the participants selected healthcare professionals through sponsored advertisements on social networks and that most participants could become patients of these professionals (Table 2). Promoting posts on Instagram involves paying to showcase photos and videos to a larger audience, including people who are not followers. It can be an effective way to increase visibility and reach potential patients. These responses present an optimistic scenario for the use of advertising by healthcare professionals.

Another way for professionals to showcase themselves on social networks is through content production. However, exposure on social networks by healthcare professionals requires good judgment. The content produced can be sensitive to viewers and may involve health vulnerabilities present in hospital environments and day-to-day practice. Therefore, it is crucial for professionals to stay up to date on the guidelines of their respective professional councils, ensuring ethical and responsible content sharing to maintain the credibility and integrity of healthcare services offered on social networks platforms. Ethical guidelines for healthcare professionals regarding postings on social networks may vary by country and specific professional board. Additionally, there is the responsibility of social networks for user-posted content. These networks have policies and terms of use that establish guidelines for what is allowed or not on their platforms, along with moderation, where they monitor user-posted content and may remove or restrict posts that violate their policies (such as misinformation, hate speech, harassment, and other issues). Pressure for social networks to take greater responsibility for content management on their platforms has been growing.

According to Morais and Brito,^25^ content creators should be attentive to followers’ needs, as they are not only consumers of the content but also of the marketing promoted by the page. The findings of this research indicate that reliability is highly valued (Figure 3), as it is believed to build trust and credibility among the audience. Similarly, the emphasis on expertise can provide authoritative and evidence-based information. The results highlight the significance of producing easy-to-understand and informative health content on social networks (Figure 4). By catering to audience preferences and educational needs, healthcare professionals and content creators can play a vital role in disseminating accurate and accessible health information, thereby contributing to improved health literacy and well-being of the population.

## Conclusion

In summary, this study revealed that social networks can influence the process of finding and selecting health professionals. Social networks, particularly Instagram, were one of the most important ways to find healthcare professionals, after recommendations from friends/relatives and other professionals. Reliability and expertise were the most valued characteristics of health content creators. Additionally, content that explains health topics in an easy-to-understand manner was the most attractive topic to survey participants.
